# Sociodemographic inequalities in multimorbidity and lifestyle risk factors among young adults: a national population-based study

**DOI:** 10.3389/fpubh.2025.1645486

**Published:** 2025-09-10

**Authors:** Mirta Mara Mendonça Tavares, Luciana Bertoldi Nucci, André Luiz Monezi Andrade, Carla Cristina Enes

**Affiliations:** ^1^Postgraduate Program in Health Sciences, Pontifical Catholic University of Campinas, Campinas, São Paulo, Brazil; ^2^Postgraduate Program in Psychology, Pontifical Catholic University of Campinas, Campinas, São Paulo, Brazil

**Keywords:** multimorbidity, noncommunicable diseases, lifestyle, socioeconomic factors, health surveys

## Abstract

**Background:**

Multimorbidity is an increasing worldwide concern, disproportionately impacting disadvantaged populations. Existing studies have mainly focused on older adults, leaving a gap in understanding how sociodemographic inequalities influence the prevalence and risk factors for multimorbidity among younger individuals. This study aims to explore sociodemographic disparities in the prevalence of multimorbidity and its behavioral risk factors among young Brazilian adults.

**Methods:**

This cross-sectional study utilized data from the 2019 National Health Survey, involving a representative sample of individuals aged 20–50 years (*n* = 48,890). Sociodemographic variables (such as gender, age, race, education, socioeconomic status, marital status, health insurance, and region of residence) and behavioral factors (smoking, alcohol use, physical activity, and eating habits) were examined in relation to multimorbidity. Logistic regression models were used to explore the associations between the selected variables and multimorbidity.

**Results:**

Of the 48,890 participants, 18% reported multimorbidity. Sociodemographic disparities were identified, with higher prevalence among women, older adults, individuals with lower education levels, and those with health insurance. Conversely, participants from the North, Northeast, and Central-West regions were associated with lower prevalence rates. The prevalence of multiple behavioral risk factors (four or more) was greater among those with lower education, lower socioeconomic status, and residents of the South and North regions. Older and married individuals exhibited a lower prevalence of concurrent risk factors.

**Conclusion:**

Sociodemographic factors such as age, gender, education, marital status, health insurance, and region were associated to multimorbidity and the co-occurrence of multiple behavioral risk factors. These disparities highlight the need for policies to reduce modifiable risk factors and promote equitable healthcare access, especially for socioeconomically disadvantaged groups.

## 1 Introduction

Sociodemographic inequalities in multimorbidity and its risk factors refer to differences in the prevalence and management of multiple chronic conditions across various population groups, shaped by factors like socioeconomic status, ethnicity, age, and occupational status. Multimorbidity is defined as having two or more chronic medical conditions at the same time and is associated to a range of adverse health outcomes, including lower quality of life, decreased functionality, and a higher risk of premature death compared to people without multimorbidity (1, 2).

Patients with multimorbidity encounter several healthcare challenges, including the need for multiple specialists, the difficulty of managing polypharmacy, and longer hospital stays—all of which lead to increased healthcare utilization and costs (1, 2). Socioeconomic disparities intensify the difficulties associated with multimorbidity, especially among marginalized racial and ethnic groups (1–3), influencing not only individual health outcomes but also broader public health results (4, 5). The prevalence of multimorbidity varies widely across different age groups, with estimates showing a baseline rate of 2.6% at age 19, rising to 35% by age 55 (6). Over the past 20 years, the occurrence of multimorbidity has increased across all age groups, with younger individuals showing higher rates than previous generations at the same age (6).

Recent studies show that people from lower socioeconomic backgrounds develop multimorbidity 10–15 years earlier than those who are more affluent, emphasizing a remarkable gap in health equity (7). Additionally, lifestyle choices including smoking and dietary habits, are key risk factors that worsen the prevalence of multimorbidity in these populations (6, 8). Gender differences further complicate the situation, as women often have higher rates of certain chronic conditions, particularly in older age groups, which interact with social determinants to affect health outcomes differently across genders (4, 9).

In the Brazilian context characterized by major regional inequalities and notable structural limitations in the healthcare system, understanding the impact of sociodemographic disparities on multimorbidity is increasingly important. While international studies highlight a clear relationship between multimorbidity and sociodemographic factors, few focus specifically on young adults in Brazil, where such inequalities can be even more pronounced. Therefore, the objective of this study was to examine how sociodemographic factors such as gender, age, education, socioeconomic status, marital status, health insurance, and region are associated with the prevalence of multimorbidity and the clustering of behavioral risk factors among Brazilian adults aged 20–50 years. The findings aim to inform public policies and targeted interventions to reduce health disparities and improve equity in healthcare access. We hypothesized that sociodemographic factors—including gender, age, education, socioeconomic status, and health insurance coverage would be associated to the prevalence of multimorbidity among young Brazilian adults.

## 2 Methods

### 2.1 Data source and participants

We used cross-sectional data from the 2019 *Pesquisa Nacional de Saúde* (PNS, National Health Survey), a nationally representative household survey conducted by the Brazilian Ministry of Health and the *Instituto Brasileiro de Geografia e Estatística* (IBGE, Brazilian Institute of Geography and Statistics). The survey aimed to evaluate health conditions, lifestyles, and the utilization of health services among individuals aged 15 years or older in Brazil.

The PNS used a complex, multistage cluster sampling strategy in three phases: census sectors served as the primary sampling units, households as the secondary units, and residents aged 15 years or older as the tertiary units. A total of 100,541 households were selected, with one resident randomly chosen from each for an individual interview; of these, 94,114 agreed to participate, resulting in a final response rate of 93.6%. For this study, we included 48,890 adults aged 20–50 years who completed the individual questionnaire. Because the data are self-reported, we excluded cases with incomplete information on the main variables.

### 2.2 Outcome variables

#### 2.2.1 Multimorbidity

We defined multimorbidity as the presence of two or more chronic diseases in an individual. The chronic diseases that the interviewees self-reported a medical diagnosis for—and which were used to identify multimorbidity—were: (i) hypertension, (ii) diabetes, (iii) high cholesterol, (iv) heart diseases (such as heart attack, angina, heart failure, or others), (v) asthma or asthmatic bronchitis, (vi) stroke, (vii) arthritis or rheumatism, (viii) chronic back problems, (ix) depression, (x) respiratory diseases (such as pulmonary emphysema, chronic bronchitis, or COPD), (xi) cancer, (xii) work-related musculoskeletal diseases, and (xiii) chronic renal failure.

#### 2.2.2 The simultaneity of risk factors

These variables were calculated based on the total number of risk factors, which were grouped as zero, one to three, and four or more risk factors (simultaneity) to examine co-occurrence. We considered data from four key domains to be the main modifiable risk factors for chronic diseases (10): smoking, alcohol consumption, physical inactivity, and unhealthy eating behaviors. Dichotomous variables indicate all: (i) current smoking, (ii) problematic alcohol use (≥5 drinks on a single occasion) (11), (iii) physical inactivity (<150 min of light or moderate activity weekly) (12), (iv) excessive screen time (>3 h daily), (v) frequent alcohol consumption (≥6 times per week), (vi) insufficient fruit/juice and vegetable intake (<25 servings weekly) (13), (vii) regular consumption of sweets (≥5 days weekly), (viii) regular intake of soft drinks or artificial juices (≥5 times weekly) (14), (ix) regular consumption of beans (≥5 times weekly), (x) replacing lunch with a quick snack (≥5 times weekly).

### 2.3 Independent variables

#### 2.3.1 Sociodemographic variables

Information was collected on the following sociodemographic variables: gender (men and women), age (20–29, 30–39, 40–50), race/skin color (White, Black, Yellow, Brown, and Indigenous), education level: (i) illiterate or incomplete elementary education, (ii) completed elementary and incomplete high school, (iii) completed high school and incomplete higher education, (iv) completed higher education; socioeconomic level (low, middle, and high) (15), marital status (single, married, separated/divorced/widowed), health insurance coverage (yes/no), and region of residence (North, Northeast, Midwest, Southeast, and South). The sociodemographic variables selected were based on their relevance to the research question and evidence from previous studies indicating their association with multimorbidity.

### 2.4 Statistical analysis

Logistic regression was employed to evaluate the relationship between sociodemographic variables and the occurrence of multimorbidity outcome. The association between the clustering of lifestyle-related risk factors and the independent variables was examined using a multinomial logistic regression model, reported as the prevalence ratio (PR) and 95% confidence interval (95% CI). Analyses were performed with SAS Studio, version 3.81, considering the sample design, and a *p*-value threshold of 0.05 was adopted.

### 2.5 Ethics statement

The Brazilian National Ethics Research Committee (CONEP) approved the PNS 2019 in August 2019 (n. 3.529.376). All the participants signed an informed consent form.

## 3 Results

Approximately one in five adults aged 20–50 reported having multimorbidity (Table 1). Regarding demographic characteristics, women made up more than half of the sample. The largest age group was 30–39, and most participants identified as mixed race. Most participants had finished high school or had some college education, and over 70% lacked private health insurance. Additionally, more than half were single and belonged to a lower socioeconomic group. Geographically, the largest portion of participants resided in the Southeast region, followed by the Northeast. Multimorbidity was more prevalent among individuals aged 40–50, women compared to men, and those with lower educational attainment. It was also more common among people who were separated, divorced, or widowed, as well as among individuals with health insurance. Considering regional differences, the South had the highest prevalence, followed by the Southeast.

**Table 1:** Sociodemographic characteristics of the study population.

Variables	*n*	(95% CI)
Age groups (years)		
20–29	12,961	30.4 (29.6–31.3)
30–39	17,832	35.5 (34.7–36.3)
40–50	18,097	34.1 (33.3–34.8)
Gender		
Men	23,848	48.5 (47.8–49.3)
Women	25,042	51.5 (50.7–52.2)
Race/skin color		
White	16,196	40.3 (39.5–41.2)
Black	5,755	11.9 (11.4–12.5)
Yellow	353	0.8 (0.6–0.9)
Brown	26,196	46.4 (45.6–47.2)
Indigenous	390	0.6 (0.4–0.7)
Education level		
Illiterate or incomplete elementary education	13,316	23.3 (22.6–24.0)
Completed elementary school and incomplete high school	7,334	15.1 (14.6–15.7)
Completed high school and incomplete higher education	19,300	43.1 (42.2–43.9)
Completed higher education	8,940	18.5 (17.7–19.3)
Socioeconomic level		
Low	30,987	53.4 (52.4–54.4)
Medium	14,410	36.6 (35.7–37.4)
High	3,493	10.0 (9.4–10.7)
Marital status		
Single	27,256	53.1 (52.2–54)
Married	18,082	40.3 (39.4–41.2)
Separated/divorced/widowed	3,552	6.6 (6.2–6.9)
Health insurance coverage		
Yes	10,688	26.6 (25.6–27.5)
No	38,202	73.4 (72.5–74.4)
Region of residence		
North	10,355	8.7 (8.3–9.1)
Northeast	17,182	27.2 (26.4–27.9)
Midwest	5,791	8.0 (7.6–8.4)
Southeast	9,843	42.0 (40.8–43.1)
South	5,719	14.2 (13.6–14.8)

Source: Instituto Brasileiro de Geografia e Estatística (27). CI, Confidence interval.

The simultaneity of risk factors for chronic diseases based on sociodemographic characteristics is described in Table 2. Most individuals reported having one to three risk factors for non-communicable diseases (NCDs). The prevalence of having four or more risk factors was higher among younger people. This pattern was also more evident among men, those who had completed elementary school or some high school, and individuals from higher socioeconomic groups. Additionally, it was more common among single people, those without health insurance, and residents of the South region of the country.

**Table 2:** Simultaneity of risk factors for chronic diseases according to sociodemographic characteristics in the Brazilian adult population.

Variables	Risk factors for NCDs			*p*-value
	0	1–3	≥4	
	% (95% CI)			
Age groups (years)				<0.001
20–29	0.8 (0.5–1.2)	49.9 (48.4–51.4)	49.3 (47.8–50.8)	
30–39	1.5 (1.1–1.8)	58.5 (57.2–59.8)	40.0 (38.7–41.3)	
40–50	1.4 (1.1–1.7)	65.0 (63.6–66.4)	33.6 (32.2–35.0)	
Gender				<0.001
Men	1.1 (0.8–1.4)	55.6 (54.5–56.7)	43.3 (42.2–44.4)	
Women	1.4 (1.1–1.6)	60.5 (59.3–61.6)	38.1 (37.0–39.3)	
Race/skin color				0.139
White	1.4 (1.1–1.7)	58.0 (56.7–59.3)	40.6 (39.3–41.9)	
Black	1.1 (0.6–1.6)	55.5 (53.3–57.7)	43.4 (41.2–45.6)	
Yellow	2.6 (0.0–5.7)	54.6 (46.3–63.0)	42.8 (34.5–51.1)	
Brown	1.2 (0.9–1.4)	59.0 (57.9–60.1)	39.8 (38.7–41.0)	
Indigenous	0.9 (0.0–2.2)	51.6 (42.1–61.1)	47.5 (38.0–57.1)	
Education level				<0.001
Illiterate or incomplete elementary education	0.6 (0.4–0.8)	60.1 (58.6–61.5)	39.3 (37.9–40.8)	
Completed elementary school and incomplete high school	0.8 (0.5–1.1)	52.6 (50.6–54.7)	46.6 (44.6–48.6)	
Completed high school and incomplete higher education	1.2 (0.9–1.5)	57.0 (55.8–58.3)	41.8 (40.5–43.0)	
Completed higher education	2.6 (2.0–3.3)	62.5 (60.7–64.4)	34.8 (33.0–36.7)	
Socioeconomic level				<0.001
Low	0.7 (0.5–0.8)	57.7 (56.7–58.7)	41.6 (40.6–42.6)	
Medium	1.7 (1.3–2.1)	58.2 (56.8–59.6)	40.1 (38.7–41.5)	
High	2.6 (1.5–3.7)	59.9 (57.2–62.7)	37.5 (34.8–40.1)	
Marital status				<0.001
Single	0.9 (0.7–1.2)	52.6 (51.6–53.7)	46.4 (45.3–47.5)	
Married	1.7 (1.4–2.0)	64.9 (63.6–66.2)	33.4 (32.1–34.7)	
Separated/divorced/widowed	1.1 (0.6–1.6)	60.5 (57.8–63.2)	38.4 (35.7–41.1)	

CI, Confidence interval.

Table 3 presents the relationship between multimorbidity and sociodemographic factors. The multivariate analysis, individuals aged 40–50 were associated with a 4.12 times higher prevalence of multimorbidity than those aged 20–29. Women also had a higher prevalence of multimorbidity than men (PR = 2.06; 95% CI: 1.88–2.56).

**Table 3:** Association between the occurrence of multimorbidity and sociodemographic variables.

Variables	PR (crude)	95% CI	PR (adjusted)	95% CI
Age groups (years)				
20–29	REF		REF	
30–39	2.06	1.79–2.38	1.96*	1.69–2.26
40–50	4.63*	4.03–5.32	4.12*	3.56–4.77
Gender				
Men	REF		REF	
Women	2.06*	1.89–2.52	2.06*	1.88–2.26
Race/skin color				
White	REF		REF	
Black	0.89	0.77–1.03	0.97	0.83–1.13
Brown	0.86*	0.78–0.94	0.98	0.88–1.09
Indigenous	0.95	0.59–1.52	1.13	0.69–1.85
Yellow	0.68	0.37–1.26	0.75	0.39–1.42
Education level				
Illiterate or incomplete elementary education	1.32*	1.16–1.50	1.56*	1.33–1.82
Completed elementary school and incomplete high school	0.90	0.77–1.04	1.28*	1.09–1.52
Completed high school and incomplete higher education	0.80*	0.71–0.90	1.10	0.96–1.25
Completed higher education	REF		REF	
Socioeconomic level				
Low	0.93	0.79–1.09	–	–
Medium	1.03	0.86–1.22	–	–
High	REF		–	–
Marital status				
Single	REF		REF	
Married	1.50*	1.37–1.64	1.01	0.92–1.11
Separated/divorced/widowed	2.12*	1.83–2.46	1.14	0.97–1.34
Health insurance coverage				
Yes	1.31*	1.20–1.43	1.36*	1.21–1.52
No	REF		REF	
Region of residence				
North	0.66*	0.58–0.74	0.71*	0.63–0.81
Northeast	0.76*	0.68–0.84	0.77*	0.69–0.86
Midwest	0.77*	0.66–0.89	0.79*	0.68–0.92
South	1.03	0.90–1.16	1.04	0.90–1.19
Southeast	REF		REF	

Max-rescaled *R*-square: 0.113; AIC intercept only: 82048711; AIC intercept and covariates: 75682329. Likelihood ratio test for Global Null Hypothesis: *F* value = 194.07; Numerator degrees of freedom = 16.36; Denominator degrees of freedom = 6812.15; *p*-value < 0.001. Second-order Rao–Scott design correction 0.0387 applied to the Likelihood Ratio test. *p < 0.05. CI, confidence interval; PR, prevalence ratio; REF, reference category.

Regarding education, individuals without formal education or with incomplete elementary education had a prevalence 1.56 times higher compared to those with completed higher education. In contrast, those with completed elementary education or incomplete high school had a prevalence 1.28 times higher. Health insurance coverage was also associated with a higher prevalence of multimorbidity, with insured individuals showing a 1.36 times higher prevalence. Finally, geographically, residents of the North, Northeast, and Central-West regions had lower prevalences of multimorbidity than those in the Southeast region.

Table 4 shows the association between behavioral risk factors for multimorbidity and sociodemographic variables. A higher prevalence of having 1–3 behavioral risk factors was seen among individuals with lower education levels, including those who are illiterate or have incomplete elementary education, and those with finished elementary or incomplete high school (PR = 1.88)—compared to those with completed higher education. Lower socioeconomic status also correlated with a higher prevalence of the outcome (PR = 2.12) compared to individuals in upper classes. Geographically, residents of the Southeast and South regions had higher prevalences than those in the Midwest.

**Table 4:** Association between the simultaneity of behavioral risk factors for multimorbidity and sociodemographic variables.

Variables	1–3 vs. 0				≥4 vs. 0			
	Crude PR (95% CI)	*p*-value	Adjusted PR (95% CI)	*p*-value	Crude PR (95% CI)	*p*-value	Adjusted PR (95% CI)	*p*-value
Age groups (years)								
20–29	REF		REF		REF		REF	
30–39	0.67 (0.40–1.108)	0.125	0.75 (0.46–1.23)	0.254	0.46 (2.28) – (0.75)	0.074	0.59 (0.36–0.97)	0.036
40–50	0.77 (0.48–1.24)	0.736	0.82 (0.51–1.30)	0.398	0.40 (0.25–0.65)	0.001	0.50 (3.13–0.79)	0.003
Gender								
Men	REF		REF				REF	
Women	0.88 (0.65–1.20)	0.427	0.91 (0.67–1.24)	0.533	0.71 (0.52–8.98)	0.034	0.76 (0.55–1.04)	0.084
Race/skin color								
White	REF				REF			
Black	1.23 (0.71–2.11)	0.459	–		1.37 (0.79–2.36)	0.261	–	
Brown	1.22 (0.88–1.69)	0.228	–		1.18 (0.85–1.63)	0.328	–	
Indigenous	1.41 (0.31–6.46)	0.660	–		1.85 (0.40–8.63)	0.433	–	
Yellow	0.50 (0.15–1.67)	0.261	–		0.56 (0.17–1.88)	0.349	–	
Education level								
Completed higher education	REF		REF		REF		REF	
Illiterate or incomplete elementary education	4.47 (2.90–6.88)	<0.001	2.76 (1.59–4.78)	<0.001	5.25 (3.38–8.14)	<0.001	3.54 (2.02–6.18)	<0.001
Completed elementary school and incomplete high school	2.84 (1.73–4.67)	<0.001	1.88 (1.08–3.28)	0.026	4.52 (2.74–7.43)	<0.001	2.75 (1.57–4.80)	0.000
Completed high school and incomplete higher education	2.01 (1.39–2.91)	<0.001	1.53 (0.96–2.41)	0.070	2.64 (1.82–3.84)	<0.001	1.84 (1.16–2.93)	0.010
Socioeconomic level								
High	REF		REF		REF		REF	
Medium	1.49 (0.92–2.41)	0.108	1.24 (0.75–2.07)	0.403	1.64 (1.01–2.66)	0.044	1.27 (0.76–2.11)	0.364
Low	3.62 (2.24–5.85)	<0.001	2.12 (1.19–3.77)	0.011	4.18 (2.59–6.74)	<0.001	2.20 (1.24–3.91)	0.007
Marital status								
Single	REF		REF		REF		REF	
Married	0.68 (0.49–0.94)	0.021	0.88 (0.63–1.24)	0.471	0.40 (0.28–0.55)	<0.001	0.57 (0. 41–0.81)	0.001
Separated/divorced/widowed	0.96 (0.55–1.68)	0.891	1.19 (0.68–2.06)	0.545	0.69 (0.40–1.21)	0.194	1.01 (0.58–1.76)	0.966
Health insurance								
Yes	0.52 (0.37–0.73)	0.002	1.02 (0.69–1.50)	0.919	0.47 (0.33–0.65)	<0.001	1.00 (0.68–1.47)	0.997
No	REF		REF		REF		REF	
Region of the country								
Southeast	REF		REF		REF		REF	
North	3.42 (2.26–5.18)	<0.001	2.53 (1.67–3.83)	<0.001	3.03 (1.99–4.61)	<0.001	2.01 (1.32–3.06)	0.001
Northeast	1.78 (1.21–2.60)	0.003	1.30 (0.89–1.91)	0.172	1.19 (0.81–1.76)	0.376	0.78 (0.53–1.16)	0.223
Midwest	1.02 (0.69–1.52)	0.913	1.00 (0.67–1.49)	0.997	0.93 (0.62–1.40)	0.728	0.87 (0.57–1.30)	0.492
South	1.70 (1.10–2.61)	0.016	1.74 (1.13–2.68)	0.012	2.09 (1.35–3.24)	0.001	2.11 (1.36–3.28)	<0.001

Max-rescaled *R*-square: 0.061; AIC intercept only: 136328811; AIC intercept and covariates: 131879322. Likelihood ratio test for Global Null Hypothesis: *F* value = 78.08; Numerator degrees of freedom = 28.88; Denominator degrees of freedom = 214,512; *p*-value < 0.001. Second-order Rao–Scott design correction 0.0390 applied to the Likelihood Ratio test. CI, confidence interval; PR, Prevalence ratio; REF, reference category.

Age and marital status were associated with having four or more risk factors, alongside education level, socioeconomic status, and region. People aged 30–39 (PR = 0.59) and 40–50 (PR = 0.50) were associated with a lower prevalence compared to younger groups. Married individuals also had a lower prevalence of the outcome than those who were single.

## 4 Discussion

This study examined sociodemographic inequalities in multimorbidity and its behavioral risk factors among young Brazilian adults. The main findings showed meaningful disparities in the prevalence of multimorbidity and related risk factors in Brazilians aged 50 years and younger, with about one in five living with the condition. Women, older individuals, and those with lower education levels were more vulnerable. Moreover, behavioral risk factors were more common among people with less education, lower socioeconomic status, and those living in the Southern and Southeastern regions.

These data align with other studies that have shown the crucial role of sociodemographic variables—including age, gender, marital status, and educational levelin the prevalence and progression of multimorbidity (16–18). For instance, older adults are more likely to face multiple chronic conditions due to natural aging and the cumulative impact of health-related behaviors over time. Additionally, some conditions like depression, arthritis, and autoimmune diseases are more common in women, while men are more often affected by higher rates of cardiovascular diseases, diabetes, and substance use disorders (2, 19). These gender-specific patterns in disease occurrence influence overall health outcomes and how healthcare services are utilized. The higher prevalence of multimorbidity among women can be associated with both biological and social factors, such as longer female life expectancy and greater use of health services by women, which may result in more frequent diagnoses of multiple conditions (20, 21).

Regarding educational levels, a higher prevalence of multimorbidity and multiple risk factors was observed among individuals with lower levels of education. Income, education, and occupational status contribute to different access to healthcare, nutrition, and healthy living conditions, all of which are crucial in shaping an individual’s health trajectory (2, 22). This association underscores the importance of education in encouraging healthy behaviors and managing chronic diseases (23).

Considering socioeconomic levels, individuals with lower socioeconomic status may be more likely to engage in unhealthy behaviors due to limited access to resources and information (22). Furthermore, the accumulation of chronic conditions is often associated with lifestyles that do not promote health, making interventions targeting these behaviors essential for reducing the impact of multimorbidity (22, 24). Private health insurance was also associated with a higher prevalence of multimorbidity, which may indicate detection bias, as individuals with health insurance have greater access to diagnoses and treatments. This finding aligns with other studies (21, 25), which highlight the influence of access to health services on the prevalence of multimorbidity. Moreover, reliance on self-reported data may further amplify this detection bias, as individuals with greater access to healthcare and health literacy are more likely to receive and report formal diagnoses. In contrast, those with lower education or no insurance may underreport conditions, contributing to differential misclassification.

Regional disparities in health outcomes are evident in Brazil, with the South showing the highest prevalence of multimorbidity and co-occurrence of risk factors, followed by the Southeast. This pattern reflects the country’s deeply rooted socioeconomic inequalities. The South and Southeast regions, being the most economically developed, have greater availability of and access to healthcare services, which may lead to higher diagnosis rates of chronic conditions. In contrast, less developed regions might experience underdiagnosis due to limited healthcare infrastructure and barriers to medical care. These differences highlight the importance of healthcare access as a key factor in explaining the regional variations.

Given this scenario, addressing sociodemographic inequalities in multimorbidity and its behavioral risk factors requires comprehensive public health policies aimed at reducing socioeconomic disparities and ensuring universal access to healthcare. Effective interventions must focus on key health determinants like obesity, poverty, and limited healthcare access (21). Promoting policies that encourage healthy eating and regular physical activity, along with strengthening public healthcare systems—such as Brazil’s Sistema Único de Saúde (SUS)—are vital for better health outcomes in various populations. Although SUS has greatly expanded healthcare access since it was established, regional disparities in coverage and quality still exist. To close these gaps, ongoing investment, improved resource allocation, and strategies emphasizing equity and integrated care are essential.

To reduce these inequalities, investing in health promotion and NCD prevention programs that address social factors is essential. Educational campaigns should focus on healthy eating, physical activity, and reducing sedentary behavior—especially in Brazil’s North and Northeast regions, which still face major structural challenges. Additionally, policies that increase healthcare access and strengthen primary care are vital to lower barriers to early diagnosis and improve the overall management of multimorbidity.

The simultaneous presence of behavioral risk factors among groups with lower education levels calls for an intersectoral approach that encourages educational and socioeconomic policies. Initiatives such as increasing access to healthy foods and regulating ultra-processed products can enhance public health efforts. A notable example is the federal government’s definition of the New Basic Food Basket, which includes recommendations from the Food Guide for the Brazilian Population, highlighting proper and healthy eating (26). Additionally, the federal government announced a reduction to 15% in the limit for processed and ultra-processed foods on public school menus starting in 2025, aiming to provide healthier diets for students. The inclusion of sugary drinks among the products to be taxed, recently approved in Brazil, is also an example of a tax policy intended to decrease the consumption of these beverages and increase access to healthier foods.

The most vulnerable populations identified in this study require targeted interventions, such as programs aimed at low-income women and initiatives focused on older adults in poor regions. Additionally, it is essential to strengthen policies that promote healthy lifestyles and address inequalities in healthcare access, considering Brazil’s cultural and regional specificities.

Another important aspect of reducing health inequalities is adopting multisectoral and global approaches that promote social justice and combine different areas of action. It is relevant to understand that health is not just a biomedical or technical issue, but one that involves social, economic, and political factors, requiring broad and coordinated strategies.

This study has several limitations. First, the cross-sectional design prevents establishing direct causal relationships between sociodemographic variables, behavioral risk factors, and multimorbidity. The associations identified represent statistical correlations observed at a specific point in time and do not allow us to determine, for instance, whether low educational attainment precedes the development of multiple chronic conditions or whether the presence of these conditions negatively impacts educational or economic outcomes. Future longitudinal studies are necessary to confirm these associations and provide strong evidence for preventive measures. Another important issue is that we used data from a secondary source; in some cases, the way the original variables were collected did not allow for more detailed analysis, especially concerning lifestyle variables.

Additionally, all health conditions and behavioral risk factors were self-reported by participants, which may introduce recall bias and social desirability bias. These biases can result in underreporting or overreporting of health-related behaviors and diagnoses, especially among individuals with limited access to healthcare, no health insurance, or lower educational levels. Consequently, these groups may face underdiagnosis or have limited health literacy, leading to differential misclassification. These biases should be considered when interpreting the results and designing future research.

Finally, lifestyle encompasses more than the behaviors examined here, and factors such as sleep duration, drug use, and variations in physical activity were not included. Future studies should adopt longitudinal designs to understand causal relationships better and include gender minorities and adolescents, as these groups may have specific characteristics that are not yet well understood within the epidemiological framework. Additionally, more detailed regional investigations could capture local particularities, considering Brazil’s diversity in infrastructure, healthcare access, and cultural contexts. The main findings of this study highlight the importance of addressing sociodemographic inequalities in healthcare access and promoting equitable health policies to reduce the burden of multimorbidity among vulnerable populations.

Further analyses are recommended to assess the effectiveness of current public strategies targeting NCDs. This includes the “Programa Previne Brasil,” a primary care funding model set by Ordinance No. 2,979/2019, which will be replaced in 2024 by the “Piso da Atenção Primária à Saúde (PAPS),” along with other health promotion initiatives in primary care. Such studies can uncover implementation gaps and help refine these strategies to ensure they serve all populations equitably.

In conclusion, our findings suggest important associations between sociodemographic factors such as age, gender, education, marital status, health insurance, and region to multimorbidity and the co-occurrence of multiple behavioral risk factors, which reflect underlying socioeconomic disparities. These results highlight the need for public health strategies aimed at reducing these inequalities. These disparities highlight the need for policies to reduce modifiable risk factors and promote equitable healthcare access, especially for socioeconomically disadvantaged groups.

## Data Availability

The original contributions presented in the study are included in the article/supplementary material, further inquiries can be directed to the corresponding author.
